# Pomegranate Extracts as Dual Regulators of Angiogenesis: A Systematic Review of Preclinical Evidence in Cancer and Chronic Wound Healing

**DOI:** 10.1002/mnfr.70060

**Published:** 2025-04-08

**Authors:** Anna Eleonora Caprifico, Gianpiero Calabrese, Riccardo Tornese, Anna Montefusco, Rocco Placì, Teodoro Semeraro, Miriana Durante, Monica De Caroli, Marcello Salvatore Lenucci

**Affiliations:** ^1^ School of Allied Health Sciences, Faculty of Health & Life Sciences De Montfort University The Gateway Leicester UK; ^2^ School of Life Sciences Pharmacy and Chemistry Kingston University London Penrhyn Road London UK; ^3^ Dipartimento di Scienze e Tecnologie Biologiche ed Ambientali (DiSTeBA) Università del Salento, Via Prov.le Lecce Monteroni Lecce Italy; ^4^ Research Institute on Terrestrial Ecosystems (IRET‐URT Lecce), National Research Council of Italy (CNR) Campus Ecotekne Lecce Italy; ^5^ Istituto di Scienze delle Produzioni Alimentari (ISPA)‐CNR Via Prov.le Lecce‐Monteroni Lecce Italy

**Keywords:** angiogenesis, cancer, chronic wound healing, pomegranate extracts, vascular endothelium growth factor

## Abstract

Angiogenesis plays a critical role in both tumor progression and wound healing. This systematic review investigates the effect of pomegranate (*Punica granatum*, PG) extracts as both anti‐ and pro‐angiogenic agents in preclinical models of cancer and chronic wound healing (CWH), respectively. Following PRISMA guidelines, 14 studies (10 cancer, 4 CWH) were identified from PubMed, Scopus, and Google Scholar databases. In cancer models, PG extracts (juice, peel extract, seed oil) reduced vascular endothelial growth factor (VEGF) expression, and endothelial tube formation across multiple cancer types, with concomitant decrease in matrix metalloproteinases and inflammatory mediators. Conversely, in CWH models, topical PG peel extract applications enhanced VEGF expression and wound closure in diabetic and burn injuries. This dual angiogenic effect appears mechanistically linked to peroxisome proliferator‐activated receptor signaling pathways and synergistic interactions among PG polyphenols, particularly ellagitannins. Assessment of study quality revealed generally low risk of bias across in vitro studies, while animal studies demonstrated variable methodological rigor. Despite promising preclinical evidence, standardization of extraction methods, exploration of molecular mechanisms, and translation to clinical investigations remain critical research priorities. This comprehensive analysis validates PG extracts as a promising therapeutic strategy for both inhibiting pathological angiogenesis in cancer and promoting beneficial angiogenesis in CWH.

## Graphical abstract

Pomegranate extracts contain polyphenols with: anti‐angiogenic properties inhibiting angiogenesis in Cancer resulting in decreased cancer growth; pro‐angiogenic properties promoting angiogenesis in chronic wound healing hence increasing the healing process

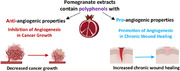

**Abbreviations**
ADSC: adipose‐derived stem cells
Bcl: B‐cell lymphoma
BPH: benign prostatic hypertrophy
CD: cluster of differentiation
COX: cyclooxygenase
CWH: chronic wound healing
EA: ellagic acid
ECM: extracellular matrix
EGF: epidermal growth factor
HBMEC: human brain microvasculature endothelial cells
HUVECs: human umbilical vein endothelial cells
IL: interleukin
MCF: Mitching Cancer Foundation
MM: multiple myeloma
MMP: matrix metalloproteinase
NOS: nitric oxide synthases
PDGF: platelet‐derived growth factor
PG: *Punica granatum*
PGE: prostaglandin
PICO: population intervention comparison outcome
PPE: pomegranate peel extract
PPAR: peroxisome proliferator‐activated receptor
PRISMA: preferred reporting items for systematic reviews and meta‐analysis
PSO: pomegranate seed oil
QUIN: quality assessment tool for in vitro studies
R: receptor
RoB: risk of bias
SYRCLE: systematic review center for laboratory animal experimentation
TGF: tumor growth factor
TNBC: triple‐negative breast cancer
TNF: tumor necrosis factor
VEGF: vascular endothelial growth factor

## 1 Introduction

A massive network of arteries, arterioles, veins, venules, and capillaries supplies oxygen and nutrients and removes metabolic waste products from each tissue and organ. This network is generated by angiogenesis, a process involved in several physiological conditions including embryogenesis, placenta formation, wound healing, and the menstrual cycle [1]. Angiogenesis is a process by which new blood vessels develop from pre‐existing vasculature and, under physiological conditions, is tightly regulated by a molecular balance between pro‐ and anti‐angiogenic factors.

Pro‐angiogenic factors are endogenous stimulators whose expression increases upon tissue metabolic demand (e.g., oxygen level) and correlate with pathological neovascularization. When endothelial cells receive a stimulating message, they secrete special enzymes including matrix metalloproteinases (MMPs) and heparinase that digest the extracellular matrix (ECM), forming breaks in the tight connections between endothelial cells so that the latter can move and advance to generate new capillary tubes [2]. In this process, angiogenic growth factors play a key role. These include vascular endothelial growth factors (VEGFs), platelet‐derived growth factors (PDGFs), cluster of differentiation 34 (CD34) [3], basic fibroblast growth factor, and other angiopoietin molecules; the VEGF family ligands are the most relevant in angiogenesis and include VEGF‐A, VEGF‐B, VEGF‐C, VEGF‐D, and the placental growth factor [4]. To perform their biological function, the VEGF family members bind to their receptors localized on vascular endothelial cells, for instance, VEGF‐A binds to VEGF‐receptor(R)‐1 with a higher affinity than VEGFR‐2, though the binding to the latter results in most angiogenic activities on the tumor endothelium [4]. Indeed, within VEGF family members, VEGF‐A is a key regulator of endothelial cell sprouting, migration, vasodilation, and permeability; and therefore, it is upregulated in tumors [4]. In rapidly growing tumors, severe hypoxia (i.e., low oxygen level) stabilizes hypoxia‐inducible factors that trigger the transcription of VEGF‐A. The latter binds to VEGFR‐2 inducing its auto‐phosphorylation and activating signal transduction pathways such as phosphatidylinositol 3‐kinase, phospholipase C‐gamma, protein kinase B, and mitogen‐activated protein kinase. These pathways are involved in many pro‐tumorigenic processes, including proliferation, cell survival, and migration [4]. The concept of targeting angiogenesis for cancer therapy emerged in the 1970s, leading to the development of anti‐angiogenic agents designed to hinder the formation of new blood vessels [5]. For instance, plasma VEGF has a prognostic value in melanoma and its reduction through anti‐angiogenic therapy inhibits cancer cell proliferation [6].

Conversely, anti‐angiogenic factors (e.g., thrombospondins, angiostatin, and endostatin) inhibit angiogenesis and help maintain the quiescence of the vascular system [7]. Decreased angiogenesis can also be due to the degradation of pro‐angiogenic factors by proteases, a feature of the wound‐healing process under some pathological conditions (e.g., diabetic or infected burn wounds): usually, wound healing needs VEGF and PDGF to trigger the growth of blood vessels and produce a new ECM [8]; new capillaries are embedded in granulation tissue, acting as a matrix for proliferating endothelial cells, fibroblasts, and new collagen [9]. In diabetic or infected burn wounds, impaired granulation can lead to the development of chronic wounds (a wound taking longer than 12 weeks to heal) [10]. In chronic wound healing (CWH), growth factors such as VEGF, tumor growth factor (TGF)‐β, and dermal ECM components are degraded by the high levels of proteases, making dermal reconstitution significantly constrained [11, 12].

Emerging research highlights the critical role of diet, macronutrients, and bioactive compounds in modulating inflammation, oxidative stress, and metabolic pathways. These are key contributors to the progression of chronic diseases such as hypertension, diabetes, cardiovascular disease, cancer, and metabolic syndrome, which are leading causes of morbidity and mortality worldwide. Among dietary macronutrients, conjugated linoleic acids have been shown to influence body composition, oxidative stress, and immune function, contributing to the regulation of inflammatory responses and metabolic homeostasis [13]. Similarly, pycnogenol, a plant‐derived flavonoid complex, has demonstrated potent anti‐inflammatory and antioxidant effects, with potential therapeutic applications in conditions such as hypertension, diabetes, and atherosclerosis [14]. Beyond macronutrients, polyphenolic compounds found in various fruits, vegetables, and plant extracts, including resveratrol (red grapes), epigallocatechin gallate (green tea), and curcumin (turmeric) have shown promise in modulating angiogenesis and tumor progression [15]. In particular, pomegranate (*Punica granatum*, PG) distinguishes itself through its diverse polyphenolic composition, which includes punicalagins, ellagic acid, and anthocyanins. These compounds not only influence multiple angiogenic pathways but also provide strong antioxidant and anti‐inflammatory effects; for instance, PG extracts can suppress tumor angiogenesis and cancer progression in pancreatic and colon cancer models [16], making PG a uniquely versatile candidate for therapeutic applications [17].

PG juice is rich in water, macro and microelements, carbohydrates, pectin, organic acids, and other compounds such as fatty acids, amino acids, and tocopherols. Its intense red pigmentation is mainly due to polyphenols including anthocyanins and ellagitannins [18]. The kernel oil (pomegranate seed oil, PSO) is rich in punicic acid, in lower amount tocopherols, and both steroidal and non‐steroidal estrogenic phytochemicals [19]. The peel is the non‐edible part of the fruit, treated as a waste, albeit PG peel extract (PPE) contains polyphenols, particularly hydrolyzable tannins, such as ellagitannins and gallotannins, which are relevant for many pharmaceutical applications [20, 21]. Ellagitannins include punicalagin, punicalin, and ellagic acid (EA). These exert potent antioxidant and anti‐inflammatory properties, crucial in the context of cancer and CWH therapies. Indeed, when macrophages and neutrophils infiltrate the microenvironment, they release pro‐inflammatory cytokines, chemokines, and reactive oxygen species leading to chronic inflammation and oxidative stress [22]. The anti‐inflammatory properties of polyphenols rely on the immune cells expressing many types of receptors that bind to polyphenols, activating different signaling pathways that regulate the immune response [23]. For instance, epigallocatechin gallate (the most efficient polyphenol in green tea for cancer prevention [24]) binds to the zeta chain‐associated protein on T cells inhibiting the T cell‐induced pathway mediated by CD3 in leukemic cells [23]. Therefore, polyphenols may exert strong anti‐cancer activities through immune‐mediated processes [23]. By interfering with the immune response, polyphenols are indirectly involved in the angiogenesis process: for instance, EA can inhibit the VEGFR‐2 receptor activation [25] and may act as either an anti‐ or pro‐angiogenic agent, according to the conditions [15]. However, a recent study [26] showed that the combination of punicalagin, punicalin, and EA in the PPE enhanced the anti‐inflammatory response over isolated single molecules. Synergistic effects may arise from the interaction of individual bioactive components of PG acting on common molecular targets. These targets encompass a wide range of signaling molecules, receptors, and transcription factors including cyclooxygenase‐2 (COX‐2), nuclear factor‐kappa B, and peroxisome proliferator‐activated receptors (PPAR‐α and PPAR‐γ) [26].

The literature review provided by Tornese et al. [17] explored the anti‐angiogenic effects of PG extracts. In contrast, this review takes a broader approach, analyzing PG extract's therapeutic effects associated with both angiogenesis inhibition and promotion, in the context of cancer and CWH, respectively. This review adheres to the preferred reporting items for systematic reviews and meta‐analysis (PRISMA) guidelines [27] to ensure a rigorous and transparent evaluation of the literature. The main findings from the studies, including the effects of PG extracts on angiogenic molecules and inflammatory pathways, are highlighted. Finally, gaps in existing research are identified, and recommendations for future study directions are provided to support the translation of these findings into clinical applications.

## 2 Literature Search Methodology

### 2.1 Database Search

A comprehensive literature search was conducted using scientific databases (Google Scholar, Scopus, and PubMed). Key terms used for searching in titles, abstracts, and keywords were as follow: “pomegranate,” “*Punica granatum*,” “VEGF,” “vascular endothelial growth factor,” “angiogenesis,” and “angiogenetic.” Boolean operators including AND and OR were utilized to refine the search results; for example, “pomegranate” OR “*Punica granatum*.” The search strategy aimed to encompass a wide array of studies focusing on PG extracts employed as therapeutic agents in cancer and CWH, ensuring a thorough review of the existing evidence. This approach provides a robust foundation for the analysis of the preclinical efficacy and therapeutic applications of PG extracts.

### 2.2 Inclusion Criteria

Studies were chosen according to several criteria. They were required to be published in peer‐reviewed journals and focus on preclinical evaluations (either in vitro or animal models) due to the absence of clinical trials. Included studies had to employ whole PG extracts (not purified compounds, e.g., purified EA or PG extracts in combination with other drugs). Studies had to focus exclusively on PG extracts as anti‐ or pro‐angiogenic agents for cancer and CWH therapies. Only studies providing detailed intervention, controls, and outcome information were included. Studies published in the last decade were prioritized to ensure the findings' relevance to current research and advancements. Only studies published in English were considered to maintain consistency and guarantee accurate interpretation of the data.

### 2.3 Exclusion Criteria

Studies were excluded if they were not available in English, consisted of unpublished data, reviews, book chapters, newsletters, magazines, or letters to the editor; used PG extracts for other therapeutic purposes rather than cancer or CWH; and assessed angiogenic activities in non‐cancerous models of neovascularization. Finally, articles were excluded if full text was not available or did not satisfy preclinical study criteria. This exclusion procedure ensured that only relevant and accessible research was included, maintaining a high level of evidence and enabling precise analysis and interpretation of the data.

### 2.4 Study Selection and Design

Two authors gathered information from the selected papers, resolving any disagreement by a third author. Following the initial search, articles were screened for relevance. Full‐text articles were reviewed to extract the following detailed information on: population, interventions, comparison, and outcomes (PICO criteria, Table 1).

**TABLE 1:** Parameters of the PICO framework.

Parameter	Criteria
Population	In vitro and animal studies
Intervention	PG extracts (juice, PPE, PSO, or by‐products)
Comparison	Non‐treated group
Outcomes	Effects of PG extracts on angiogenic and related inflammatory factors

Abbreviations: PG, pomegranate; PPE, pomegranate peel extract; PSO, pomegranate seed oil.

Only experimental studies were included in the study design of this systematic review. In vitro studies examined endothelial cell behavior using angiogenesis‐related assays including tube formation assays, wound healing assays, proliferation tests, and VEGF quantification. Animal studies utilized preclinical tumor models to evaluate angiogenesis, employing methods including wound closure rate, VEGF or epidermal growth factor (EGF) levels in the tissue wound lysate. The PG extracts were administered through oral or topical routes with doses ranging from micrograms to milligrams per kilogram of body weight. The primary outcomes measured included changes in angiogenesis markers such as VEGF, CD34, MMPs, and PPARs, while secondary outcomes focused on inflammatory and oxidative stress markers relevant to angiogenesis, including prostaglandin (PGE2), interleukin (IL)‐6, and tumor necrosis factor (TNF)‐α.

### 2.5 Quality Assessment

The quality of the included studies was systematically evaluated. The methodological rigor of each study was assessed by examining the study design, the appropriateness of models and controls, and the clarity in defining interventions and outcomes. Sample sizes were analyzed to ensure the reliability of conclusions, and the thoroughness of data reporting was evaluated to confirm the transparency and accuracy of the presented results. The risk of bias was assessed using the quality assessment tool for in vitro studies (QUIN tool) [28] and the Systematic Review Center for Laboratory Animal Experimentation (SYRCLE)’s risk of bias (RoB) tool for animal research [29]. The QUIN tool evaluated 12 domains and categorized them into high, unclear, and low risk of bias. Studies scoring above 70% were classified as having low risk of bias; those scoring between 60% and 70% were considered to have a medium risk of bias; and those scoring between 50% and 60% were classified as high risk. The SYRCLE's RoB tool for animal research assessed 10 criteria where “Y” indicated a low risk of bias, “N” signified a high risk, and “U” denoted an unclear risk. Particular emphasis was placed on the studies’ potential for translation into clinical applications, acknowledging the need for rigorous validation in animal models before progressing to clinical trials. By systematically applying these criteria, this review ensured a comprehensive and objective evaluation of the quality of the included studies.

## 3 Literature Search PRISMA Findings and Quality Overview of Included Studies

**FIGURE 1:**
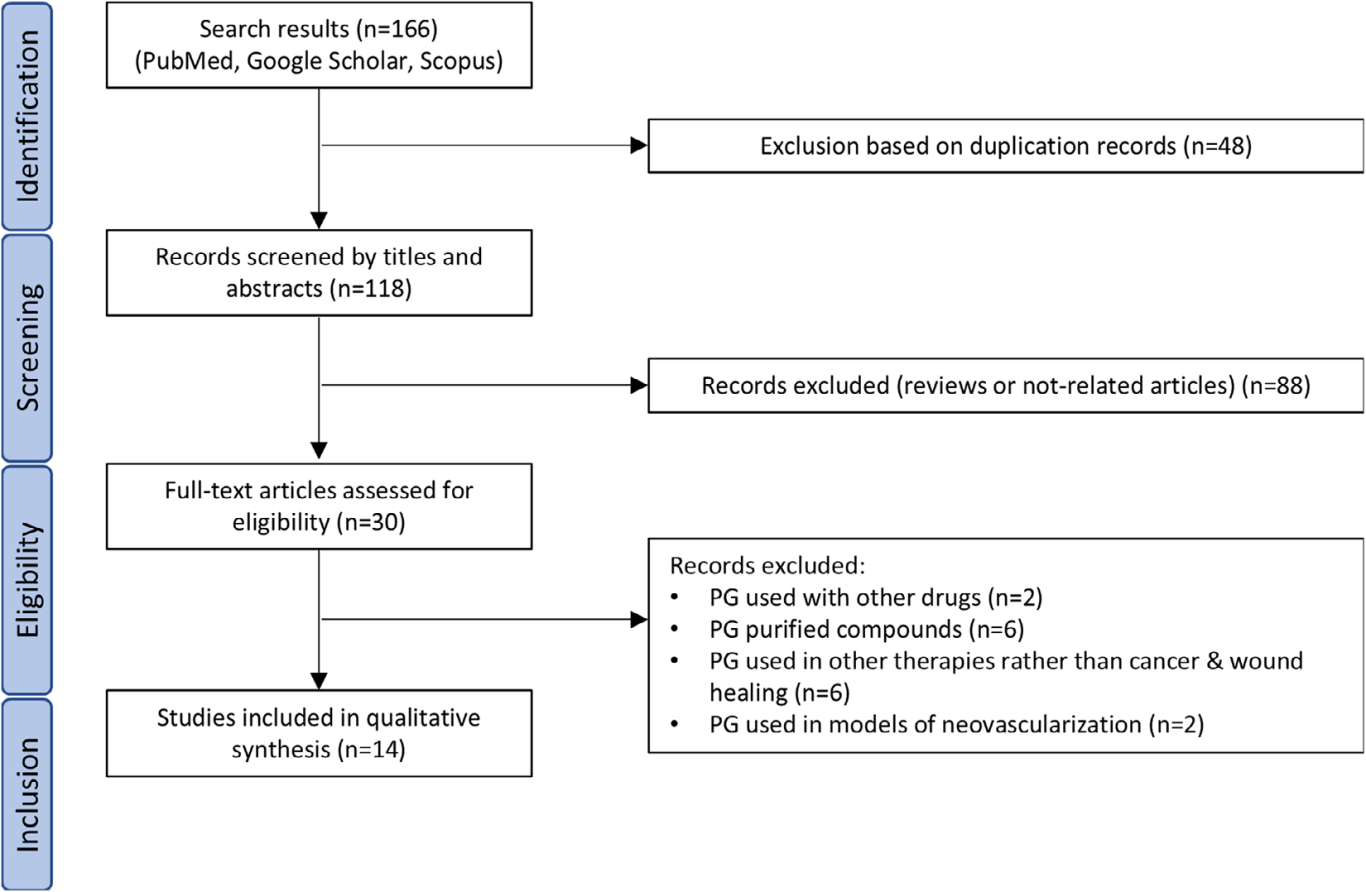
PRISMA flowchart of screening and inclusion of eligible articles. PG indicates pomegranate.

To offer a clear summary of the study selection process for this review, the PRISMA diagram [27] (Figure 1) depicts the flow of information through the various stages, from initial identification of studies to final inclusion in the systematic review. Records were determined from databases (*n* = 166) and, before screening, duplicate records (*n* = 48) were removed. This left a total of 118 records for screening by title and abstract. Out of those, 88 records were excluded because they were reviews or not‐related articles. Reports were then sought for retrieval (*n* = 30), assessed for eligibility, and excluded based on following specific criteria: studies using PG extracts used in combination with other drugs (*n* = 2), purified PG compounds (*n* = 6), PG extracts used in other therapies rather than cancer and CWH (*n* = 6), and PG extracts used in models of neovascularization (*n* = 2). Finally, 14 studies were included in the review. These studies covered a variety of cancers, including melanoma, breast, and prostate cancer (*n* = 10) along with burn and diabetic CWH models (*n* = 4).

### 3.1 Pomegranate Extracts as Anti‐Angiogenic Agents in Cancer Therapy

According to the inclusion criteria, 10 studies assessing PG extracts as anti‐angiogenic agents in cancer therapy were included in the systematic review (Table 2). Hernawati and Irmawati [30] showed that PG whole extract (75 mg/kg/day, for 4 weeks) was more effective than the control or isolated EA in decreasing the expression of VEGF mRNA and inducing the apoptosis of oral cancer cells in Swiss Balb/c mice. El‐Kott [31] showed that, following oral administration of PG juice extract (10% w/v) to lung cancer‐induced Swiss albino mice for 16 weeks, the expression of CD34 (a marker of angiogenesis [3]) was significantly decreased in the lung tissue, suggesting that the neovascularization was inhibited along with cancer cell proliferation. Tibullo et al. [32] assessed the antiangiogenic effects of PG juice (3% and 6%) up to 48 h, in the context of multiple myeloma (MM), using human brain microvascular endothelial cells and several MM cell lines; an inhibitory effect on the tube formation and endothelial cell migration was recorded along with a decreased expression in several angiogenic genes (e.g., MMP2 and VEGF). Inhibition of MM cell proliferation was caused by the ability of PG juice to upregulate PPAR‐γ mRNA (*p* < 0.0001) [32].

**TABLE 2:** Anti‐angiogenic activity of pomegranate extracts in cancer therapy.

Intervention	Population	Dose	Duration	Primary outcome	Secondary outcome	Ref.
Whole extract	Oral cancer‐bearing mice	75 mg/kg/day	4 weeks	Reduced VEGF mRNA expression	Increased apoptosis of cancer cells	[30]
Juice	Lung cancer‐bearing mice	10% w/v	16 weeks	Reduced expression of CD34, hence microvessel density	Reduced cell proliferation and oxidative stress	[31]
Juice	HBMEC and MM cells	3%, 6%	24, 48 h	Inhibition of tube formation length and branch points. Reduced endothelial cell migration, mRNA VEGF expression, MMP2, and other angiogenic genes	Inhibition of cancer cell proliferation by upregulation of PPARγ mRNA	[32]
PPE	Prostate cancer‐bearing mice	80, 112, or 187 mg/kg/day	17 days	Reduced VEGF serum levels at the highest dose	Inhibition of tumor growth. Increased TNF‐α levels in serum	[33]
PPE	HepG2 cells	20 µg/mL	24 h	Reduced VEGF and MMP9 mRNA expression	Decreased cell viability and COX‐2	[34]
Black PPE	HUVEC and B16F10 cells	200–400 µg/mL	24 h	Reduced size tube, length, and number of junctions of the tubes; inhibition of VEGF mRNA expression and secretion.	Toxicity toward B16F10 but not toward HUVECs.	[35]
PPE	TNBC cells	12.5–1000 µg/mL	72 h	Downregulation of VEGF, MMP9, and fibronectin.	Increased apoptotic cells and decreased cell migration.	[36]
PSO	Co‐culture: MCF‐7 cells/ADSC	100 µg/mL	24 h	Reduced VEGF mRNA expression	Inhibition of cell proliferation by reducing Bcl‐2 while upregulating Bax/caspase 3.	[37]
PSO	MCF‐7 cells	0.6 µg/mL	24 h	Reduced VEGF mRNA expression	Decreased cell proliferation and other pro‐inflammatory cytokines (e.g., IL‐6, TNF‐α)	[38]
By‐product's extract	H5V cells; BPH cells	1 mg/mL; 0.01 mg/mL	48 h	Reduced VEGF levels in H5V and BPH cells	Decreased level of nitrite/nitrate, PGE2, IL‐6	[39]

Abbreviations: ADSC, adipose‐derived stem cells; Bcl, B cell lymphoma; BPH, benign prostatic hypertrophy; CD, cluster of differentiation; COX, cyclooxygenase; HBMEC, human brain microvascular endothelial cells; HUVEC, human umbilical vein endothelial cells; IL, interleukin; MCF, Michigan cancer foundation; MM, multiple myeloma; MMP, matrix metalloproteinase; PGE, prostaglandin; PPE, pomegranate peel extract; PPAR, peroxisome proliferator‐activated receptor; PSO, pomegranate seed oil; TNF, tumor necrosis factor; TNBC, triple‐negative breast cancer; VEGF, vascular endothelium growth factor.

Ma et al. [33] administered PPE (80, 112, or 187 mg/kg/day) for 17 days to subcutaneous xenograft of human prostate cancer cells. The serum VEGF levels decreased at the highest dosage of PPE compared to the control, leading to tumor growth inhibition and increased TNF‐α levels in serum. In another study, the anti‐tumorigenic effects of PPE (20 µg/mL for 24 h) were evaluated in a human hepatocellular carcinoma cell line against a normal liver cell line [34]. The expression of genes related to cancer progression and angiogenesis (e.g., VEGF and MMP9) decreased in a concentration‐dependent manner compared to normal cells, showing that PPE possessed a selective effect on cancer cell lines [34]. Dana and Refiee [35] assessed the ability of black PPE (200, 300, and 400 µg/mL) to reduce angiogenesis in human umbilical vein endothelial cells (HUVECs) and a melanoma cell line (B16F10). The VEGF mRNA expression and secretion in the culture medium of treated cells significantly dropped in a dose‐dependent manner, suggesting the ability of PPE to reduce angiogenesis [35]. Ahmadiankia et al. [36] showed that the treatment of a triple‐negative breast cancer (TNBC) cell line with PPE (up to 1000 µg/mL) for 72 h decreased the VEGF mRNA expression. Other genes involved in the metastasis of TNBC including MMP9 were also decreased in a dose‐dependent manner compared to the control (*p* < 0.01). Similarly, Moradi‐Gharibvand et al. [37] treated Michigan Cancer Foundation (MCF)‐7 cells in co‐culture with adipose‐derived stem cells with PSO (100 µg/mL) for 24 h. Results showed that the gene expression of VEGF and B cell lymphoma (Bcl)‐2 was downregulated while the expression of Bax and caspase 3 was upregulated compared to the control. Similarly, Costantini et al. [38] treated MCF‐7 cells with PSO (up to 0.6 µg/mL) for 24 h and showed that the levels of VEGF and several pro‐inflammatory cytokines significantly decreased with increasing doses of the extracts. Finally, Consoli et al. [39] investigated the anti‐angiogenic properties of PG's by‐product extracts (exhausted peels, membranes, and arils) in endothelial cells, H5V, incubated with the culture medium of benign prostatic hypertrophy (BPH) cells, to mimic angiogenesis in the BPH microenvironment. The tube morphology, length, and branch points decreased in a dose‐dependent manner (up to 1 mg/mL) of extract. In addition, PGE2, IL‐6, nitrite/nitrate, and VEGF levels were significantly reduced in H5V cells incubated with the highest concentration of the extract.

### 3.2 Pomegranate Extracts as Pro‐Angiogenic Agents in CWH

According to the inclusion criteria, four studies employing PG extracts as pro‐angiogenic agents in CWH were included in the systematic review (Table 3). Zhang et al. [40] tested the effects of PPE on the expression of VEGF‐A in second‐degree burn in the skin tissue of minipigs, simulating human‐burned skin. PPE was topically administered and embedded in a gel matrix (5% w/w) for 35 days; an increased healing rate (expressed by the wound closure rate) and VEGF level were recorded, suggesting a therapeutic effect of PPE on burn wound healing. Karim et al. [41] employed Saudi PPE gel (5% w/w) on the wounds of diabetic Sprague Dawley rats. Results showed that the highest VEGF mRNA expression in the wound tissue lysate occurred after 14 days of treatment. Accordingly, an increased TGF‐β1, EGF, and collagen production were recorded along with low nitric oxide synthase (NOS) activity. A similar result was reported by Karim et al. [42] applying Saudi PPE gel (5% w/w) to the wound of diabetic Wistar rats. Following 21 days of treatment, the wound increased its closure rates, VEGF mRNA, and neovascularization compared to the control. Collagen production, EGF, and IL‐4 levels increased while IL‐1β, IL‐17, and IL‐10 levels decreased compared to the control. Finally, Scappaticci et al. [43] employed a spray formulation of PPE to treat infected skin wounds of diabetic rats for 14 days. Results showed a significant increase in wound healing rate compared to the control. Especially, an increased inflammatory infiltration was recorded after 2 and 7 days of treatment, suggesting that the pro‐inflammatory response triggered the wound‐healing process [43].

**TABLE 3:** Pro‐angiogenic activity of pomegranate extracts in chronic wound healing.

Intervention	Population	Dose	Duration	Primary outcome	Secondary outcome	Ref.
PPE	Burned skin of minipigs	Gel matrix (5% w/w)	35 days	Increased VEGF‐A mRNA expression.	Increased TGF‐β1 mRNA expression.	[40]
Saudi PPE	Diabetics rats	Gel matrix (5% w/w)	14/21 days	Increased VEGF mRNA expression.	Increased TGF‐β1, EGF mRNA expression, and collagen production. Low NOS activity.	[41]
Saudi PPE	Diabetics rats	Gel matrix (5% w/w)	21 days	Increased VEGF mRNA expression.	Increased collagen production, EGF, and IL‐4 levels. Decreased levels of IL‐1β, IL‐17, and IL‐10.	[42]
PPE	Diabetics rats	Spray	14 days	Increased number of blood vessels.	Inflammatory cell infiltration.	[43]

Abbreviations: EGF, epidermal growth factor; IL, interleukin; NOS, nitric oxide synthases; PG, pomegranate; PPE, pomegranate peel extract; TGF, transforming growth factor; VEGF, vascular endothelium growth factor.

### 3.3 Risk of Bias Assessment

The risk of bias within the included in vitro studies (Table 4) was assessed using established criteria such as details of the comparison group and an explanation of the methodology. Only one study [36] had a high risk of bias, while the remaining studies [32, 34, 35, 37–39] had a low risk of bias.

**TABLE 4:** Risk of bias assessment of included articles using the quality assessment tool for in vitro studies (QUIN tool).

	Studies						
Quality criteria	Tibullo et al. [32]	Basal et al. [34]	Dana et al. [35]	Ahmadiankia et al. [36]	Moradi‐Gharibvand et al. [37]	Costantini et al. [38]	Consoli et al. [39]
Clearly stated aims/objectives	1	2	1	2	1	1	1
Detailed explanation of sample size calculation	1	2	2	1	1	2	1
Detailed explanation of sampling technique	2	2	2	1	2	2	2
Details of comparison group	2	2	2	0	1	2	2
Detailed explanation of methodology	2	2	2	1	2	2	2
Operator details	2	2	0	0	2	2	2
Randomization	2	2	2	2	2	2	2
Method of measurement of outcome	1	2	2	2	2	2	2
Outcome assessor details	2	2	0	0	2	2	2
Blinding	2	2	0	0	2	2	2
Statistical analysis	2	2	2	2	2	2	2
Presentation of results	2	2	2	2	2	2	2
Total score	21	24	17	13	21	23	22
Percentage (%)	88	100	71	55	88	96	92

The risk of bias was also assessed within the included animal studies (Table 5) using established criteria, such as random sequence generation, allocation concealment, blinding of outcome assessors, and selective outcome reporting. All investigations adhered to complete data reporting standards and appeared free of selective outcome reporting. Baseline characteristics of the animals, such as body weight, pre‐surgery mechanical, and thermal pain thresholds, were reported in all included articles. Two studies employed blinding for researchers (investigators experimenting) and utilized blinding of outcome assessment to evaluate the animals’ responses [40, 42].

**TABLE 5:** Risk of bias assessment of included articles using the SYRCLE's RoB tool for animal research.

Quality criteria	Studies						
	Hernawati and Irmawati [30]	El‐Kott [31]	Ma et al. [33]	Zhang et al. [40]	Karim et al. [41]	Karim et al. [42]	Scappaticci et al. [43]
Sequence generation (selection bias)	U	U	U	U	U	U	U
Baseline characteristics (selection bias)	Y	Y	Y	Y	Y	Y	Y
Allocation concealment (selection bias)	U	U	Y	U	U	U	U
Random Housing (performance bias)	U	U	U	U	U	U	U
Blinding of personnel (performance bias)	U	U	U	Y	U	Y	U
Random outcome assessment (detection bias)	U	U	U	U	U	U	U
Blinding of outcome assessment (detection bias)	U	U	U	Y	U	Y	U
Incomplete outcome data (attrition bias)	Y	Y	Y	Y	Y	Y	Y
Selective outcome reporting (reporting bias)	Y	Y	Y	Y	Y	Y	Y
Other sources of bias	U	U	U	U	U	U	U

Abbreviations: U, unclear risk of bias; Y, low risk of bias.

## 4 Toxicity, Angiogenic Therapeutic Potential, and Future Research Directions of PG Extracts in Cancer and CWH Therapies

PG stands out for its ability to interfere with angiogenesis as both anti‐ [20, 21, 44–46] and pro‐angiogenic agent [47, 48, 49]. However, to the author's knowledge, this is the first systematic review comparing existing papers on the dual effects of PG extracts on angiogenesis in two major clinical conditions—cancer and CWH.

The analysis of the included studies, limited to animal and in vitro investigations, revealed no cytotoxicity, mortality, or weight loss following topical administration [41, 42]. Furthermore, following intragastric administration, no systemic toxicity was observed up to the medium dose of PPE (112 mg/kg) [33]. No clinical studies have explored PG extracts as an angiogenic agent. However, several clinical trials have evaluated the effectiveness of orally administered PG for the treatment of diabetes [50]. For instance, fresh PG juice consumption (1.5 mL/kg) in diabetic patients significantly reduced the fasting blood glucose and insulin resistance [51], along with IL‐6 levels [52]. Furthermore, the intake of PG extracts (two capsules/day for 4 weeks) significantly decreased lipid peroxidation (involved in the oxidative stress) and modulated liver enzymes in diabetic patients [53]. PG capsules were also effective in reducing lipopolysaccharide‐binding protein levels, a marker of endotoxemia, in patients affected by colorectal cancer [54]. However, Zare et al. [55] reported some clinical studies showing side effects associated with the intake of PG, more commonly including gastrointestinal and urinary problems, flu‐like symptoms, and allergic reactions. Cytotoxic concerns may hinder the translation of PG extracts into clinical applications, partly due to the extraction methods of polyphenols. Conventional methods such as maceration, Soxhlet extraction, and cold pressing often use methanol or ethanol, raising sustainability and safety concerns due to the potentially toxic solvent residues [56]. More sustainable green extraction methods, including ultrasound‐assisted, microwave‐assisted, enzyme‐assisted, hydrodynamic cavitation, and sub‐ or supercritical fluid extraction, have emerged as safer alternatives. However, none of the included studies employed these novel extraction techniques, highlighting a research gap that could impact the clinical development of PG extracts as an angiogenic agent.

The analysis revealed in vitro studies reporting a decreased neovascularization in multiple tumor models [32, 34–39]. Similarly, animal studies demonstrated that oral or intragastric administration of PG extracts led to tumor growth reduction by inhibiting angiogenesis [30, 31, 33]. In contrast, animal studies on CWH demonstrated that the topical administration of PG extracts enhanced angiogenesis by upregulating the VEGF mRNA expression [40, 41, 42, 43]. These findings suggested that PG extracts exhibit both angiogenic effects depending on the disease and mode of administration. The dual effect on angiogenesis is a well‐established feature of dietary polyphenols such as resveratrol, epigallocatechin gallate, and curcumin, which have been shown to modulate angiogenesis and inflammation in a context‐dependent manner [15, 57]. However, PG extracts are particularly rich in polyphenols, mainly hydrolyzable tannins, such as punicalin, pedunculagin, punicalagin, gallagic acid, and EA derivatives. Indeed, most included studies employed PPE or other by‐products as either anti‐angiogenic [33–36, 39] or pro‐angiogenic [40, 41, 42, 43] agents given the high polyphenol content of PPE, mainly EA. EA has been shown to inhibit the VEGF‐R2 activation of Tip cells, a specialized subset of endothelial cells that guide angiogenic sprouting and capillary formation [25, 58]. Interestingly, Hernawati and Irmawati [30] showed that the whole PG extract was more effective than isolated EA in decreasing VEGF mRNA levels and inducing apoptosis of cancer cells in vivo. This contrasts with resveratrol, which retains its bioactivity independently of other compounds. The increased bioactivity in the whole PG extract may be attributed to EA's low bioavailability and conversion into urolithins—intestinal microbial metabolites formed when EA is administered alone [59]. Additionally, other bioactive components of PG extracts may contribute to the angiogenesis‐modulating effects. For instance, galactomannan, a polysaccharide isolated from the PG peels, inhibited neovascularization in chicken embryos model [60], while commercially purified punicalagin (purity > 98%) reduced VEGFR2 activation and p21‐activated kinase 1 expression upon VEGF stimulation in HUVECs [35].

Chen and Tseng [57] suggested that several factors, including dose, pharmacokinetics, and experimental conditions, influence whether polyphenols contained in PG extracts or resveratrol act as pro‐ or anti‐angiogenic agents. The dose used in vivo for cancer therapy ranged from 75 to 187 mg/kg/day over 17 days to 16 weeks, resulting in reduced angiogenesis markers and tumor growth in oral [30], lung [31], and prostate cancers [33]. Regarding in vitro studies of hepatic and breast cancers, PG extracts (juice, PPE, and PSO) were tested at 0.4–1000 µg/mL for up to 72 h, leading to VEGF mRNA downregulation and inhibition of cancer cell proliferation [34, 36–38]. Therefore, both in vitro and in vivo studies support PG's anti‐angiogenic effects. To better simulate the tumor microenvironment, some studies co‐cultured endothelial cells with cancer cell‐conditioned medium, revealing significant reductions in tube formation upon treatment with PG extracts (juice, 6% [32]; black PPE, 400 µg/mL [35]; by‐product extracts, 1 mg/mL [39]). The anti‐angiogenic effects of PG extracts were associated with reduced oxidative stress [31]; nitrite/nitrate levels [39]; pro‐inflammatory cytokines (TNF‐α [33], COX‐2 [34], PGE2 [39]); and MMP9 [34], while simultaneously upregulating apoptotic genes (Bax, p21, and caspase 3 [34, 37]) and PPARs mRNA expression [32]. Conversely, in the CWH model, PG extracts (especially PPE) increased VEGF mRNA expression and neovascularization [40, 41, 42, 43]. The pro‐angiogenic effects were linked to TGF‐β1, EGF, and collagen production while reducing NOS activity and pro‐inflammatory cytokines [40, 41, 42]. Interestingly, Scappaticci et al. [43] found that an increased inflammatory infiltration activated the wound‐healing process in diabetic rats. Furthermore, PG extracts may modulate angiogenesis via PPARs [32]. Indeed, Dana et al. [61] demonstrated that PPE acts as a PPAR‐γ and ‐α agonist in HUVECs as blocking these pathways reversed its anti‐angiogenic effects. Similarly, Seifabadi et al. [62] showed that PPE administration in melanoma‐bearing mice reduced VEGF plasma levels by activating PPAR signaling. While PPAR‐α and PPAR‐γ inhibit angiogenesis, PPAR‐β/δ is pro‐angiogenic and plays a crucial role in wound healing [63, 64]. Future studies should investigate whether PG extracts selectively activate PPAR‐β/δ in CWH, potentially explaining its dual effect on angiogenic.

Despite these promising preclinical results, several limitations should be considered. The studies included in this review are limited to preclinical models, with no clinical trials investigating the angiogenic properties of PG extracts. While animal and in vitro models provide valuable mechanistic insights, they do not fully replicate the complexity of human pathophysiology, pharmacokinetics, and immune responses. Moreover, significant variability in study design was observed across the included studies, affecting the comparability of results. Differences in PG extract preparation methods, dosages, treatment durations, administration routes, experimental models, and animal species contribute to heterogeneity in findings, hence a meta‐analysis was not performed. This limitation highlights the need for standardized experimental conditions to compare and quantify the effects of PG extracts on angiogenesis.

In conclusion, PG extracts represent a promising therapeutic approach for modulating angiogenesis, inhibiting pathological vessel formation in cancer while promoting beneficial neovascularization in chronic wounds. The natural origin, favorable safety profile, and multi‐target mechanisms of PG extracts make them valuable candidates for further development. However, translating these preclinical findings requires addressing several critical gaps including novel extraction methods with emphasis on green technologies; elucidating molecular mechanisms behind PPAR isoform selectivity; and conducting well‐designed clinical trials to validate efficacy and safety in humans. By systematically addressing these research priorities, the therapeutic potential of PG extracts on angiogenesis offers new treatment options for both cancer and CWH.

## Ethics Statement

This article does not contain any studies with human and animal subjects performed by any of the authors.

## Conflicts of Interest

The authors declare no conflicts of interest.

### Peer Review

The peer review history for this article is available at https://www.webofscience.com/api/gateway/wos/peer-review/10.1002/mnfr.70060.

## Data Availability

Data are available on request from the authors.
